# Editorial: Antithrombotic Treatment in Transcatheter Structural Cardiac Interventions and After Cardiac Device Implantation

**DOI:** 10.3389/fcvm.2020.616638

**Published:** 2020-11-12

**Authors:** Jolanta M. Siller-Matula, Julia Mascherbauer, Christian Hengstenberg

**Affiliations:** ^1^Division of Cardiology, Department of Internal Medicine II, Medical University of Vienna, Vienna, Austria; ^2^Department of Experimental and Clinical Pharmacology, Centre for Preclinical Research and Technology, Medical University of Warsaw, Warsaw, Poland

**Keywords:** TAVR, transcatheter aortic valve implantation, mitraclip, triclip, PFO occluder device, ASD occluder device

Transcatheter procedures for structural heart diseases carry a significant thromboembolic and concomitant bleeding risk, not only during the procedure but especially during the peri- and post-procedural period. Many issues concerning the optimal peri- and post-procedural antithrombotic therapy are still under debate. Especially, the optimal antithrombotic therapy after transcatheter aortic valve replacement (TAVR) is of increasing clinical relevance as recent data have shown that transcatheter heart valve thrombosis is more frequent than initially believed (1). Moreover, the importance for optimal antithrombotic therapy is underlined by the close association between subclinical transcatheter heart valve thrombosis and related adverse events. In mitral and tricuspid valves, some achievements have been made over the past few years resulting in a fair amount of new transcatheter devices with different characteristics and implantation techniques. However, optimal antithrombotic strategies in these patients are still under investigation (2). As the use of transcatheter cardiac devices is on the rise also in low risk and younger populations (3–5), many highly important and clinically relevant aspects related to the proper use of antiplatelet and anticoagulant agents were discussed in this thematic topic.

The review by Valvo et al. puts emphasis on the risk/benefit ratio for use of antithrombotic drugs after TAVR. Patients undergoing TAVR are often at high risk for both, bleeding and ischemic/embolic events, during and after the procedure. Authors discuss the mechanisms involved in bleeding and embolic complications after TAVR and give guidance for the choice, combination, and duration of the antithrombotic drugs in different clinical scenarios.

The review article by Rosseel et al. discusses the appropriate use of patient-tailored antithrombotic therapies to prevent or treat leaflet thrombosis after TAVR. Authors of the review distinguish between clinical or subclinical TAVR thrombosis. While clinical valve thrombosis can be diagnosed by increased transvalvular gradient and heart failure symptoms or embolism, the recently described subclinical leaflet thrombosis (SLT) can only be diagnosed as an incidental finding or during a screening exam by use of multi-detector computed tomography. Whereas, clinical valve thrombosis requires antithrombotic treatment, management of SLT is still uncertain. Authors of the review give deep insight into potential implications of SLT and its treatment with a focus on patient tailored antithrombotic approach.

In their review article, Olasinska-Wisniewska and Grygier give insight into the issue of device thrombosis prevention and treatment after implantation of ASD, PFO, LAA occluders, or intra-atrial shunt device. The authors discuss in detail available evidence for preprocedural, periprocedural, and postprocedural pharmacological therapy. Furthermore, the review provides guidance for diagnosis of device associated thrombosis.

In summary, the high quality overview articles address the optimal antiplatelet and anticoagulant strategies for prevention and treatment of device-associated thrombosis. These strategies are of high clinical relevance because prompt diagnosis enables therapeutic measures and will reduce morbidity and mortality. Moreover, the topic is timely and of current interest, as transcatheter devices are entering more and more routine clinical practice. Importantly, there is still a need for future trials aiming at optimizing the net clinical benefit in those patients.

## Author Contributions

JS-M, JM, and CH drafting and revision. All authors contributed to the article and approved the submitted version.

## Conflict of Interest

The authors declare that the research was conducted in the absence of any commercial or financial relationships that could be construed as a potential conflict of interest.
